# Type 2 Diabetes and Atrial Fibrillation: Evaluating Causal and Pleiotropic Pathways Using Mendelian Randomization

**DOI:** 10.1161/JAHA.123.030298

**Published:** 2023-08-23

**Authors:** Rohin K. Reddy, Maddalena Ardissino, Fu Siong Ng

**Affiliations:** ^1^ National Heart and Lung Institute, Imperial College London London United Kingdom; ^2^ Papworth Hospital, Cambridge Biomedical Campus Cambridge United Kingdom

**Keywords:** atrial fibrillation, diabetes, type 2, genes, Mendelian randomization, Atrial Fibrillation

## Abstract

**Background:**

Observational associations between type 2 diabetes (T2D) and atrial fibrillation (AF) have been established, but causality remains undetermined. We performed Mendelian randomization (MR) to study causal effects of genetically predicted T2D on AF risk, independent of cardiometabolic risk factors.

**Methods and Results:**

Instrumental variables included 182 uncorrelated single nucleotide polymorphisms associated with T2D at genome‐wide significance (*P* <5×10^−8^). Genetic association estimates for cardiometabolic exposures were obtained from genome‐wide association studies including 188 577 individuals for low‐density lipoprotein‐C, 694 649 individuals for body mass index, and 757 601 for systolic blood pressure. Two‐sample, inverse‐variance weighted MR formed the primary analyses. The MR‐TRYX approach was used to dissect potential pleiotropic pathways, with multivariable MR performed to investigate cardiometabolic mediation. Genetically predicted T2D associated with increased AF liability in univariable MR (odds ratio [OR], 1.08 [95% CI, 1.02–1.13], *P*=0.003). Sensitivity analyses indicated potential pleiotropy, with radial MR identifying 4 outlier single nucleotide polymorphisms that were likely contributors. Phenomic scanning on MR‐base and subsequent least absolute shrinkage and selection operator regression allowed prioritization of 7 candidate traits. The outlier‐adjusted effect estimate remained consistent with the original inverse‐variance weighted estimate (OR, 1.07 [95% CI, 1.02–1.12], *P*=0.008). On multivariable MR, T2D remained associated with increased AF liability after adjustment for low‐density lipoprotein‐C and body mass index. Following adjustment for systolic blood pressure, the relationship between T2D and AF became nonsignificant (OR, 1.04 [95% CI, 0.95–1.13], *P*=0.40).

**Conclusions:**

These data provide novel genetic evidence that while T2D likely causally associates with AF, mediation via systolic blood pressure exists. Endeavoring to lower systolic blood pressure alongside achieving normoglycemia may provide particular benefit on AF risk in patients with T2D.

**Nonstandard Abbreviations and Acronyms**
DIAMANTE: Diabetes meta‐analysis of trans‐ethnic association studies
GWAS: genome‐wide association study
MAGIC: Meta‐Analyses of Glucose and Insulin‐related traits Consortium
MR: Mendelian randomization
SBP: systolic blood pressure
SPRINT: Systolic Blood Pressure Intervention Trial
T2D: type 2 diabetes

**Clinical Perspective**

**What Is New?**

- Type 2 diabetes (T2D) associates with incident atrial fibrillation (AF) in observational studies, representing a potential modifiable risk factor; however, phenotypic observational analyses are liable to confounding and reverse causality to which Mendelian randomization is robust.
- Previous Mendelian randomization investigation has not shown an association between T2D and AF, but new T2D exposure data from the current largest genome‐wide association study has recently become available: in univariable Mendelian randomization, T2D associated with AF; on multivariable Mendelian randomization, this association remained robust following adjustment for low‐density lipoprotein cholesterol levels and body mass index but was attenuated when controlling for systolic blood pressure.

**What Are the Clinical Implications?**

- This study provides novel genetic evidence to support a causal association between T2D and AF but also suggests that a mediating pathway through systolic blood pressure exists, with the key clinical implication that endeavoring to lower systolic blood pressure alongside achieving normoglycemia might provide particular benefit in reducing AF risk in patients with T2D.

The observational association between type 2 diabetes (T2D) and atrial fibrillation (AF) is well established. Meta‐analysis of 1 686 097 participants demonstrated an ≈40% increased risk of incident AF in individuals with T2D.^1^ However, given the association of T2D with dysregulated cardiometabolic phenotypes, observational effect estimates are liable to residual confounding. Thus, any possible causal pathways between T2D and AF remain unclear.

Mendelian randomization (MR) uses genetic variants as instrumental variables to study relationships between genetically predicted risk factors and outcomes. Leveraging independently and randomly inherited genetic variants renders MR more robust to confounding and reverse causality than phenotypic observational study, strengthening causal inference. Previous MR investigation has not supported a causal role of T2D on AF^2^, ^3^, ^4^; however, new data from the DIAMANTE (Diabetes Meta‐Analysis of Trans‐Ethnic Association Studies)^5^ genome‐wide association study (GWAS) have recently become available, studying the largest cohort with diabetes to date. We conducted a 2‐sample multivariable MR study to investigate causal effects of T2D on AF risk and investigated potential horizontal and vertical pleiotropic pathways.

## METHODS

### Ethical Approval, Data Availability, and Reporting

All data used are openly available and therefore this study was exempt from institutional review committee approval. All GWAS used for data sources obtained relevant participant consent and ethical approval, which have been cited appropriately. All data and supporting materials used in this study can be obtained from the GWAS cited within the article.

### Data Sources

One hundred eighty‐two uncorrelated (*r* ^2^<0.001) single nucleotide polymorphisms (SNPs) associated with T2D at genome‐wide significance (*P* <5×10^−8^) were selected as instrumental variables from the DIAMANTE GWAS, containing data from 180 834 affected individuals and 1 159 055 controls.^5^ Genetic association estimates for AF were obtained from the FinnGen consortium (r7.finngen.fi).^6^

### Horizontal and Vertical Pleiotropy

Horizontal pleiotropy refers to SNPs exerting influence on the exposure studied (T2D), which subsequently influences the outcome (AF), but also simultaneously affecting other factors (eg, blood pressure), which also influence the outcome (AF). This limits causal inference on exposure–outcome relationships, as MR effect estimates incorporate additional effects of the SNP via parallel pleiotropic pathways, rather than exclusively through the exposure under investigation, shown in Figure S1A.

Vertical pleiotropy occurs when SNPs exert influence on the exposure studied (T2D), which subsequently influence the outcome (AF), but the exposure (T2D) also causes intermediate phenotypes (eg, obesity), which additionally influences outcome (AF), shown in Figure S1B. The presence of vertical pleiotropy does not negate the presence of a causal pathway between T2D and AF, but rather identifies intermediate phenotypes responsible for part (or all) of the causal effect of T2D on AF.

### Statistical Analysis

Inverse‐variance weighted MR with multiplicative random effects formed the primary analysis method for all models, to estimate the effect of genetically predicted T2D on AF. Weighted median and MR‐Egger sensitivity analyses were used to assess for the presence of horizontal pleiotropy.

**Figure 1:**
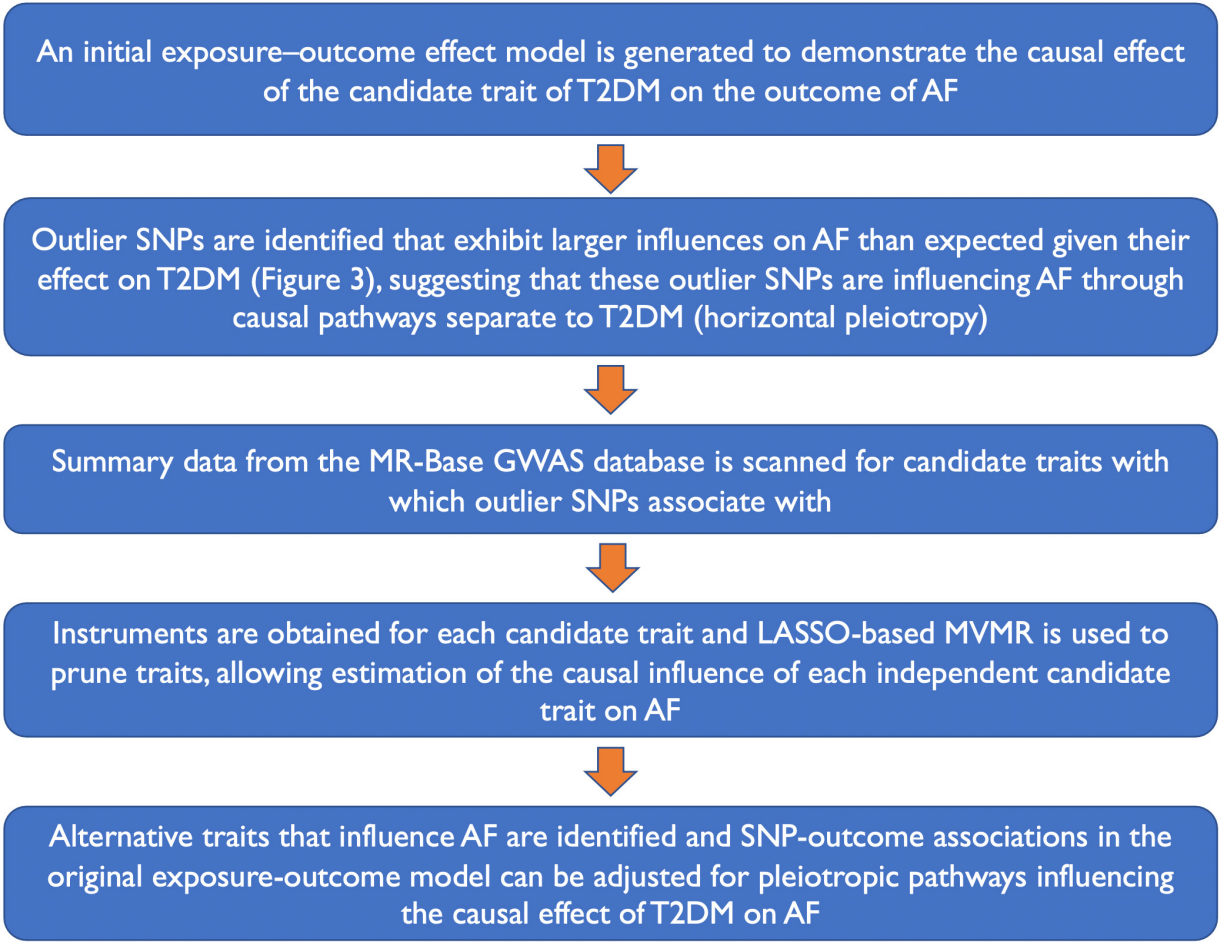
Study design framework. AF indicates atrial fibrillation; GWAS, genome‐wide association study; LASSO, least absolute shrinkage and selection operator; MR, Mendelian randomization; MVMR, multivariable Mendelian randomization; SNP, single‐nucleotide polymorphism; and T2DM, type 2 diabetes mellitus.

The MR‐TRYX approach entails identification of outlier SNPs using radial MR,^7^ and subsequently scanning the MR‐Base database to identify phenotypes that the outlier SNPs are associated with at genome‐wide significance level (candidate traits), among a list of 4835 available traits. The resultant list of candidate traits was then evaluated for association with the original exposure (T2D) bidirectionally, and with the outcome (AF) traits at a corrected false discovery rate of 0.05 on inverse‐variance weighted (IVW) MR multiplicative random effects models. For this step, the prior release data from the DIAMANTE consortium^8^ had to be utilized due to practical constraints, as the more recent GWAS analysis results^5^ are not available for analysis on MR‐Base. Candidate traits that are significant in this step are then visually inspected by authors to remove exposure‐related (eg, metformin use) or outcome‐related (eg, anticoagulant use) traits, and remove clearly duplicated overlapping phenotypes (eg, hypertension and high blood pressure). For the candidate traits carried forward, instruments excluding the original outlier SNP were obtained, and a least absolute shrinkage and selection operator‐based multivariable MR model was used to prune redundant candidate traits, and estimate the causal influence of each independent candidate trait on AF. Details of the outlier SNPs are provided in Table S1. The SNP–outcome associations in the original MR model, estimating the impact of T2D on AF, were then adjusted for the impact of the candidate traits on the outcome, and the original MR model was re‐estimated based on these adjusted SNP–outcome associations to provide an estimate of the effect of T2D on AF after accounting for horizontal pleiotropic effects. Thus, to account for horizontal pleiotropy, the MR‐TRYX^9^ framework was utilized. The study design framework is outlined in Figure 1.

Mediation analysis was performed using multivariable MR, to quantify direct effects of T2D on AF, after accounting for potential vertically pleiotropic pathways to highlight mediating factors in the relationship between T2D and AF. Putative mediators included low‐density lipoprotein cholesterol levels (LDL‐C), body mass index (BMI), and systolic blood pressure (SBP) levels. Genetic association estimates were extracted from relevant GWAS (n_LDL‐C_=188 577,^10^ n_BMI_=694 649,^11^ n_SBP_=757 601^12^). Multivariable MR analyses were performed assessing the impact of T2D on AF after conditioning on each putative mediator individually, and effect estimates from these analyses (direct effects of T2D on AF) were compared with the unadjusted IVW MR estimates (total effects of T2D on AF). The indirect effects and proportions mediated were not calculated in view of the noncollapsibility of the effect estimates for the SNP‐outcome associations. Instead, the direct and total effects were qualitatively compared with attenuations of the ORs after conditioning for a mediator taken to indicate at least partial mediation.^13^ All statistical analyses were performed using the statistical programming environment R.

## RESULTS

**Figure 2:**
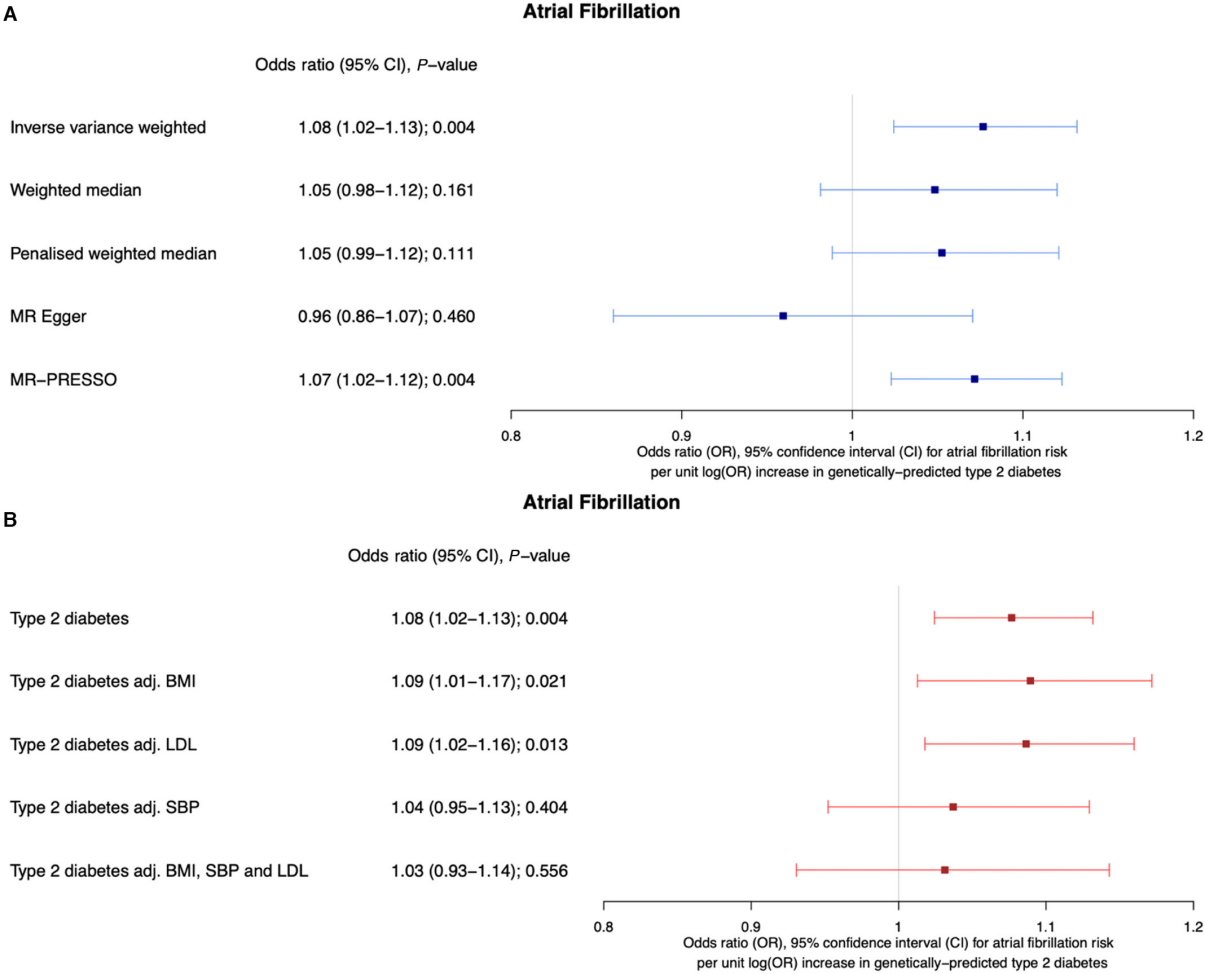
Forest plots showing Mendelian randomization (MR) summary estimates for the association between type 2 diabetes and AF. **A**, Univariable MR and sensitivity analyses for detection of pleiotropy. **B**, Multivariable MR incorporating single‐nucleotide polymorphisms responsible for pleiotropy. AF indicates atrial fibrillation; BMI, body mass index; LDL, low‐density lipoprotein; MR, Mendelian randomization; PRESSO, Pleiotropy REsidual Sum and Outlier; and SBP, systolic blood pressure.

Genetically predicted T2D was associated with increased odds of AF in the univariable IVW model (OR, 1.08 [95% CI, 1.02–1.13], *P*=0.003), though there was substantial heterogeneity in this estimate (*Q*=375.0 on 182 SNPs, *P*=1.24×10^−5^). Weighted median and MR‐Egger analyses yielded inconsistent results, indicating potential pleiotropy (OR, 1.05 [95% CI, 0.98–1.12], *P*=0.16 and OR, 0.96 [95% CI, 0.86–1.07], *P*=0.46, respectively), corroborated by a significant MR‐Egger intercept (MR‐Egger intercept 0.008, SE, 0.004, *P*=0.02) (Figure 2A).

**Figure 3:**
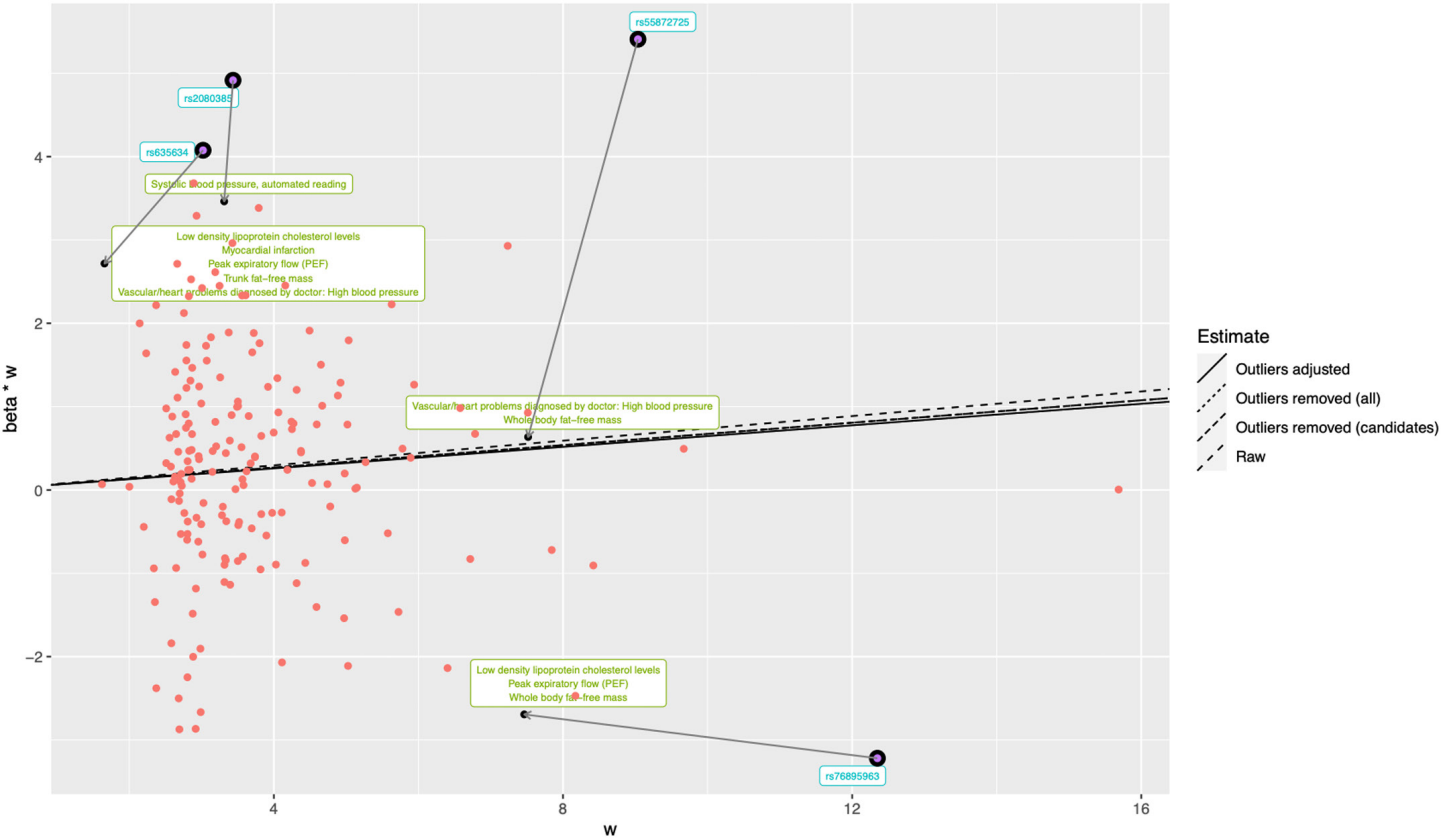
Effect estimates for type 2 diabetes on AF after adjusting the SNP effects based on the candidate traits, represented as Radial plots of MR associations. The *x* axis represents the weight (w) contributed by each SNP to the overall effect estimate, and the *y* axis represents the product of the weight (w) and the MR effect estimate (beta). The lines indicate the causal effect estimates using different outlier adjustment models. Four identified outlier SNPs are highlighted with blue labels, and the arrows depict the changes in the SNPʼs contribution to the overall causal effect estimate after adjustment for the effect of 1 or more pleiotropic traits, listed in the green text box for each SNP, on the outcome. AF indicates atrial fibrillation; MR, Mendelian randomization; SNP, single‐nucleotide polymorphism; and w, weight.

Radial MR identified 4 outlier SNPs: rs2080385, rs55872725, rs635634, and rs76895963. Among 4835 phenotypes available on MR‐Base, 189 candidate traits were associated with outliers at *P* <5×10^−8^. After manual removal of redundant and duplicate traits, 92 were putatively causal for AF at a false discovery rate *P* <0.05. Following least absolute shrinkage and selection operator regression, 7 traits were prioritized: SBP, high blood pressure, myocardial infarction, LDL‐C levels, peak expiratory flow, trunk fat‐free mass, and whole‐body fat‐free mass. Some of these were associated with multiple outlier SNPs (Table S1). We adjusted the exposure–outcome association for the detected pleiotropic pathways, minimizing the outlier effects of these variants (Figure 3). The effect estimate remained broadly consistent with the original IVW estimate (OR, 1.07 [95% CI, 1.02–1.12], *P*=0.008), as did IVW estimates when removing all outliers (OR, 1.07 [95% CI, 1.02–1.12], *P*=0.006). After adjustment for the outlier SNPs, total heterogeneity was reduced by 17.5% (*Q*=326.2), supporting a causal effect of T2D on AF.

Next, we explored potential vertical pleiotropic (mediating) pathways through LDL‐C, BMI, and SBP. On multivariable MR, T2D remained associated with increased odds of AF after adjustment for genetically predicted LDL‐C (OR, 1.09 [95% CI, 1.02–1.16], *P*=0.01) and genetically predicted BMI (OR, 1.09 [95% CI, 1.01–1.17], *P*=0.02). Following adjustment for genetically predicted SBP, the relationship between genetically predicted T2D and AF was fully attenuated (OR, 1.04 [95% CI, 0.95–1.13], *P*=0.40; Figure 2B), suggesting that increased SBP mediates at least part, if not all, of the association between T2D and AF.

## DISCUSSION

In this 2‐sample multivariable MR investigation, we took a stepwise approach towards parsing the independent effects of genetically predicted T2D on risk of AF. T2D initially associated with increased odds of AF on univariable MR, which is a novel finding. However, the presence of SNPs influencing T2D risk via alternative biological pathways was discovered. Thorough investigation of horizontal pleiotropy using Radial MR and MR‐TRYX revealed SNPs associated with T2D exerting influences on blood pressure, cholesterol, and anthropometric traits in parallel. Adjustment for these horizontally pleiotropic pathways did not substantially alter effect estimates, providing evidence to support the existence of a causal relationship between T2D and AF. However, when assessing the role of vertical pleiotropy, the association between T2D and AF remained consistent when adjusting for LDL‐C and BMI, but adjustment for SBP fully attenuated the association. This suggests that SBP at least partially, if not fully, mediates the relationship between T2D and AF.

While this study provides evidence to support a causal relationship between T2D and AF, prior MR investigation has not demonstrated an association between genetically predicted T2D and AF.^2^, ^3^, ^4^ Power in MR is dependent on the magnitude of causal effect and the proportion of variance in the risk factor explained by the instrumental variable (ie, SNPs). Power can represent a concern given that as a rule, a relatively small amount of variation in phenotypic traits is explained by genetic variants. Harati et al^2^ performed MR using a GWAS meta‐analysis from the Diabetes Genetics Replication and Meta‐analysis (DIAGRAM) consortium^14^ of 26 676 T2D cases and 132 532 control subjects for their T2D exposure and a GWAS meta‐analysis from the Meta‐Analyses of Glucose and Insulin‐related traits Consortium^15^ (MAGIC) containing variants associated with glycemic traits on up to 133 010 nondiabetic individuals. Both GWAS were undertaken in European ancestry populations. For glycemic traits, Huang et al^3^ used the MAGIC GWAS^15^ in addition to a multi‐ancestry GWAS meta‐analysis containing HbA1c measurements on up to 159 940 individuals from 82 cohorts of European, African, East Asian, and South Asian ancestry.^16^ Genetic instruments for T2D were obtained from the prior DIAMANTE consortium GWAS meta‐analysis^8^ that included 74 124 T2D cases and 824 006 controls of European ancestry. The most recent MR study that did not show a relationship between T2D and AF was performed by Kwok and Schooling,^4^ who also utilized the prior DIAMANTE consortium GWAS meta‐analysis.^8^ In this study, we leveraged the latest data release from the DIAMANTE consortium, the largest genetic cohort studying T2D to date, including data from 180 834 affected individuals and 1 159 055 controls.^5^ This analysis includes >2‐fold the number of cases compared with the prior MR study. Therefore, the most likely explanation for the discordance between previous studies and the present work is a prior lack of power to detect a statistically significant signal.

The present study is consistent with prior meta‐analysis of 1 686 097 participants demonstrating increased risk of incident AF in individuals with T2D.^1^ Indeed, in patients with T2D with diabetic retinopathy or nephropathy, a surrogate phenotype for poorly controlled T2D, incident AF was observed to be higher compared with those without microvascular complications.^17^ Similarly, optimal glycemic control in the 12 months preceding ablation was associated with reduced rates of AF recurrence following catheter ablation (2% AF recurrence for those who achieved a 10% improvement in HbA1c versus 91.1% AF recurrence for those whose HbA1c worsened in the same period).^18^ A range of putative mechanisms has been proposed to explain the relationship between T2D and AF including insulin resistance, chronic inflammation, and derangements in hemostasis, fibrinolysis, and angiogenesis.^18^, ^19^ Increases in turnover of the extracellular matrix may cause endothelial dysfunction, abnormal renin‐angiotensin‐aldosterone system activation, and atherogenesis. All of these pathophysiologic elements may contribute to structural, electrical, and autonomic remodeling with subsequent development of a pro‐arrhythmic substrate, driving propensity for AF.^18^, ^19^ Thus, there is strong biological plausibility for an association between T2D and AF, although data from adequately powered placebo‐controlled trials randomizing participants to a hypoglycemic therapeutic arm are required to definitively prove this hypothesis. This MR investigation supports Class I guideline recommendations to identify and treat risk factors, in addition to active lifestyle modification and targeted therapies to reduce AF burden.^20^

The vertically pleiotropic effect of SBP in the association between T2D and AF is clinically relevant. Genetic study previously identified beneficial effects of lower SBP on reduction of T2D risk^21^ and we highlight that a causal network between these 2 phenotypes also impacts downstream risk of AF. These results have important parallels in post hoc analyses of trials on AF risk investigating T2D medications with differential effects on SBP. Prior meta‐analysis of dipeptidyl‐peptidase‐4 inhibitors, glucagon‐like peptide‐1 receptor agonists, and sodium‐glucose co‐transporter‐2 inhibitors across cardiovascular and renal outcome trials identified that dipeptidyl‐peptidase‐4 inhibitors and glucagon‐like peptide‐1 receptor agonists had no significant effect on AF risk, whereas sodium‐glucose co‐transporter‐2 inhibitors resulted in a 19% risk reduction of 0.81 (95% CI, 0.69–0.95).^22^ An explanation for this may be that sodium‐glucose co‐transporter‐2 inhibitors produce hypoglycemia by increasing renal sodium and glucose excretion, with the resultant osmotic diuresis reducing SBP.^23^ Contrastingly, dipeptidyl‐peptidase‐4 inhibitors and glucagon‐like peptide‐1 receptor agonists primarily exert antihyperglycemic effects via the incretin pathway, which likely impact blood pressure to a lesser extent. Previous MR work has shown that SNPs associated with higher SBP associate with an increased risk of AF, with subsequent reduction of SBP through proxying antihypertensive drug targets also reducing AF risk.^24^ These data are concordant with a post hoc analysis of the SPRINT (Systolic Blood Pressure Intervention) Trial,^25^ which demonstrated a 26% lower hazard of incident AF for those randomized to the intensive (target SBP <120 mm Hg) blood pressure–lowering arm. Similarly, a prospective cohort study including 34 221 women participating in the Women's Health Study demonstrated that elevated SBP was a strong predictor of incident AF, exhibiting a graded response with increasing blood pressures.^26^ SBP was a better predictor of incident AF compared with diastolic blood pressure over 12.4 years of follow‐up.^26^ Considering the evidence for a shared pathway provided in our study, this putative blood pressure–lowering mechanism of sodium‐glucose co‐transporter‐2 inhibitors would reasonably lead to superior protection against incident AF compared with other classes that have a lesser impact on blood pressure. This is a key finding with important implications of relevance to drug‐target studies aimed at reducing burden of AF in patients with T2D.

The principal strength of this study is the MR paradigm, which strengthens causal inference through reduced vulnerability to confounding and reverse causality. This is the most comprehensive interrogation of genetic evidence underpinning the relationship between T2D and AF, and we utilized different MR approaches that vary in their assumptions to aid our inquiry. However, there are limitations. MR‐BASE has incomplete phenomic coverage, and for this reason, other pleiotropic phenotypes might be missed. Also, as current DIAMANTE candidate traits are not yet available on MR‐BASE, we relied on the prior release, which reduced power. Negative results in multivariable MR therefore may relate to low conditional power after accounting for putative gene‐mediator effects. Negative multivariable MR results should not be interpreted as confirmation or absence of mediating pathways. Finally, the data sources from which genetic association estimates were derived concentrated in mainly European ancestry individuals. Through inclusion of an ethnically homogenous study population, risk of confounding by population stratification was minimized but these results may lack generalizability on external populations. Further study is warranted in ancestrally diverse populations, given the higher prevalence and incidence of T2D in African‐Caribbean and Asian ethnic groups.^27^, ^28^, ^29^

In conclusion, our study provides novel genetic evidence to support the existence of a causal relationship between T2D and AF, but also highlights that a mediating pathway through SBP exists. The key clinical implication is that endeavoring to lower SBP alongside achieving normoglycemia might provide particular benefit in reducing AF risk in patients with T2D. Randomized trials are required to confirm these findings.

## Sources of Funding

R.K.R. has received funding from the Imperial National Institute for Health Research Biomedical Research Centre. M.A. is supported by the National Institute for Health Research (NIHR Academic Clinical Fellowship). F.S.N. is supported by the National Institute for Health Research (NIHR Biomedical Research Centre funding) and the British Heart Foundation (RG/F/22/110078 and RE/18/4/34215 for F.S.N.).

## Disclosures

None.

## Supporting information

Table S1
Figure S1
