# Supplementary material for: Type 2 Diabetes and Atrial Fibrillation: Evaluating Causal and Pleiotropic Pathways Using Mendelian Randomization
Source: J Am Heart Assoc. 2023 Aug 23;12(17):e030298. doi: 10.1161/JAHA.123.030298 (PMC10547336; doi:10.1161/JAHA.123.030298)
Supplement: Supplementary file 1 — Table S1 Figure S1 [file JAH3-12-e030298-s001.pdf]

Supplemental Material

Table S1. Candidate traits associated with both exposure and outcome prioritized on LASSO regression. LDL-C indicates low-density lipoprotein C.

Outlier SNPs	Phenotypes	Beta	SE	*p* value
rs76895963	LDL-C	-0.071	0.0074	1.00×10^{-21}
	Peak expiratory flow	0.047	0.0071	4.20×10^{-11}
	Whole body fat-free mass	0.095	0.0048	2.80×10^{-87}
rs55872725	Whole body fat-free mass	0.036	0.0013	6.19×10^{-178}
	High blood pressure	0.0062	0.00097	1.30×10^{-10}
rs635634	Myocardial infarction	0.085	0.0095	5.29×10^{-19}
	LDL-C	0.079	0.0025	2.90×10^{-225}
	Peak expiratory flow	-0.016	0.0024	4.20×10^{-12}
	High blood pressure	-0.0081	0.0012	4.90×10^{-12}
	Trunk fat-free mass	-0.0092	0.0016	8.00×10^{-9}
rs2080385	Systolic blood pressure	-0.015	0.0024	6.90×10^{-10}

Figure S1. **Directed acyclic graphs showing examples of potential horizontal and vertical pleiotropy in the relationship between T2DM and AF**.

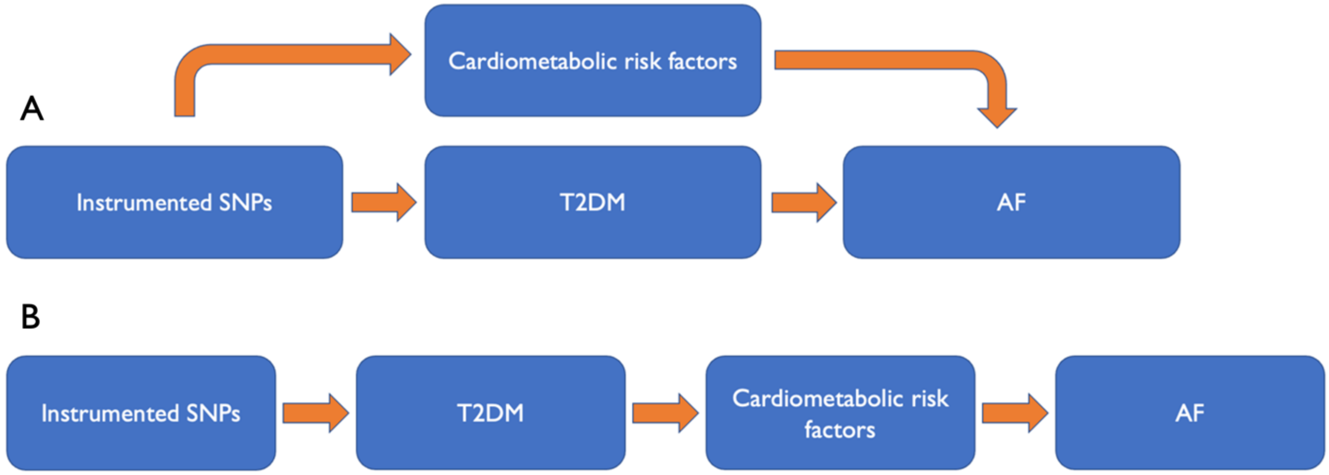

A) Horizontal pleiotropy, where genetic variants instrumented to proxy the effect of also associate with cardiometabolic risk factors. This violates the exclusion restriction assumption of MR which states genetic instruments should work only through the original proxied risk factor alone, and not via alternate parallel biologic pathways; B) Vertical pleiotropy, where genetic variants instrumented to proxy the effect of T2DM affect AF through downstream cardiometabolic risk factors, which implies mediation via these risk factors. This does not invalidate the instrumental variable assumptions underlying the MR approach. Atrial fibrillation indicates AF; MR, Mendelian randomization; SNP, single-nucleotide polymorphism; and T2DM, type 2 diabetes mellitus.
